# Spleen as an Alternative Tissue for Estimating *Tetracapsuloides bryosalmonae* Load, Prevalence and Its Relationship With Proliferative Kidney Disease in Brown Trout

**DOI:** 10.1111/jfd.14148

**Published:** 2025-06-05

**Authors:** Magnus Lauringson, Lilian Pukk, Siim Kahar, Oksana Burimski, Riho Gross, Veljo Kisand, Anti Vasemägi

**Affiliations:** ^1^ Chair of Aquaculture, Institute of Veterinary Medicine and Animal Sciences Estonian University of Life Sciences Tartu Estonia; ^2^ Institute of Technology University of Tartu Tartu Estonia; ^3^ Chair of Hydrobiology and Fisheries, Institute of Agricultural and Environmental Sciences Estonian University of Life Sciences Tartu Estonia; ^4^ Department of Aquatic Resources, Institute of Freshwater Research Swedish University of Agricultural Sciences Uppsala Sweden

**Keywords:** alternative diagnostic tissue, brown trout, fish disease, myxozoa, parasite load

## Abstract

Accurate pathogen detection is essential in fish health management and disease prevention. Pathogens often target different host tissues, and monitoring alternative target organs can provide important insights into disease progression. We evaluated the spleen as an alternative to the kidney for estimating the load and prevalence of the myxozoan parasite *Tetracapsuloides bryosalmonae* (*Tb*) and its relationships with proliferative kidney disease (PKD) in juvenile brown trout. We sampled 238 brown trout across nine rivers and quantified parasite load in both tissues using qPCR. Parasite load showed a strong positive correlation between the two tissues for both raw and log10‐transformed data (Pearson's *r* = 0.60–0.71, Spearman's *ρ* = 0.78), with the spleen exhibiting, on average, 4.1‐fold lower parasite load compared to the kidney. *Tb* was found in 191 specimens, consisting of 167 spleen and 190 kidney detections. The relationships between parasite load and PKD symptoms (renal hyperplasia and anaemia) were comparable for both tissues, and segmented regression line analysis indicated that health parameters deteriorate faster after exceeding a certain parasite load threshold. In conclusion, these results suggest that the spleen may serve as a viable alternative to the kidney for *Tb* monitoring, providing useful insights into *Tb* presence, load, and PKD progression in salmonids.

## 1 Introduction

Myxozoans are globally distributed microscopic endoparasites with complex life cycles that alternate between invertebrate hosts (such as annelids and bryozoans) and vertebrates, primarily fish, across both marine and freshwater environments (Alexander et al. 2015; Alama‐Bermejo and Holzer 2021). Certain myxozoan species, including *Myxobolus cerebralis*, *Ceratonova shasta* and *Tetracapsuloides bryosalmonae*, are notorious for causing severe diseases in fish, such as whirling disease, ceratomyxosis and proliferative kidney disease (Fontes et al. 2015). These diseases can result in high morbidity and mortality rates, severely affecting both aquaculture operations and wild fish populations (Hedrick et al. 1993; Turner et al. 2014; Bartholomew et al. 2022).

Malacosporean *Tetracapsuloides bryosalmonae* (*Tb*), the causative agent of proliferative kidney disease (PKD) in salmonids, is widespread throughout the northern hemisphere (Peribáñez et al. 1997; Mo and Jørgensen 2017; Gorgoglione et al. 2020; Svavarsdóttir et al. 2021). PKD poses a significant challenge to both wild and farmed salmonid populations (Ros et al. 2022). The parasite alternates between freshwater bryozoans, its primary invertebrate host and salmonid fish, its vertebrate host (Canning et al. 2000). The abundance of *Tb* stages infective to fish is closely linked to rising water temperatures, as increased temperatures promote bryozoan biomass and elevate the concentration of infective parasite spores in the environment (Tops et al. 2009; Hartikainen et al. 2009). Higher temperatures also drive the clinical manifestation of PKD, primarily affecting young‐of‐the‐year (YOY) salmonids during their first growing season (Okamura et al. 2011). Fish become infected when *Tb* spores penetrate the skin or gills (Longshaw et al. 2002). Once inside the vascular system, the parasite's extrasporogonic stages multiply and disseminate to the lymphoid and haematopoietic tissue, causing proliferation and immune modulation (Chilmonczyk et al. 2002; Bettge et al. 2009). Extrasporogonic stages continue to develop primarily in the kidney and spleen, though they may also be found in various other tissues and organs throughout the host (Feist and Longshaw 2006). Clinical symptoms, including pale and anaemic gills, abdominal swelling, kidney proliferation and anaemia, typically appear within 4–8 weeks of infection, especially if water temperatures exceed 15°C (Clifton‐Hadley et al. 1986). Following proliferation in the kidney interstitium, some extrasporogonic parasite stages migrate into the kidney tubules, where they persist even after clinical PKD resolves (Morris et al.  2000). During clinical PKD, parasite stages induce inflammation and proliferation of spleen tissue similar to the kidney, which serves as the primary site of parasite proliferation and spore release (Hedrick et al. 1993; Canning et al. 2002). Once sporogenesis is completed, mature spores are excreted via urine (Hedrick et al. 2004). These spores then infect bryozoans, completing the parasite's lifecycle (Morris and Adams 2007).

Spleen plays a key role in the immune system functions of fishes, particularly in processing and responding to pathogens (Zapata 2024). It acts as a secondary lymphoid organ where lymphocytes are activated and respond to infections. In addition to its role in filtering antigens, the spleen is involved in the production of immune cells and antibodies (Sayed et al. 2022). During clinical PKD, both the kidney and spleen are affected by the parasite, with pathophysiological changes like kidney enlargement and splenomegaly further highlighting the immune system's active role in combating the infection (Kotob et al. 2017).

Accurate detection and quantification of *Tb* in infected salmonids is essential for understanding disease dynamics and progression. To date, the quantification of *Tb* has been carried out using real‐time quantitative PCR (qPCR) from DNA extract from salmonid kidneys (e.g., Bettge et al. 2009; Grabner and El‐Matbouli 2009). Although *Tb* is also known to proliferate in the spleen (Canning et al. 2002), to date, no studies have compared parasite load in spleen tissue in relation to the kidney. However, compared to kidney tissue, dissecting the spleen may provide several potential advantages. For example, due to its location deep within the body cavity of the fish, sampling kidney tissue can be time‐consuming and difficult. This is especially relevant for asymptomatic PKD cases in small salmonid specimens (< 50 mm). In contrast, the spleen is easier to locate, as it is positioned inside the serosal lining of the intestine, and its distinct colouration and solid tissue structure, in contrast to the kidney, make it easier to dissect. Additionally, the distribution of *Tb* within the kidney can differ across various sections of this elongated organ during subclinical infections (Lauringson et al. 2023), adding further complexity to kidney sampling.

In this study, we assessed the potential of the spleen as an alternative host tissue for evaluating the load and prevalence of the myxozoan parasite *Tb* and its association with proliferative kidney disease in juvenile brown trout ( *Salmo trutta* ). Our specific objectives were: (i) to evaluate the prevalence of *Tb* in two tissues across multiple brown trout populations and examine the correlation between parasite load estimates in the spleen and kidney tissues; (ii) to characterise the relationships between *Tb* load in the two tissues and main symptoms of PKD, namely renal hyperplasia and anaemia; (iii) to investigate the suitability of the spleen as an alternative diagnostic tissue for estimating *Tb* load and prevalence, and to evaluate its relevance in comparing parasite load and PKD symptoms.

## 2 Materials and Methods

### 2.1 Sample Collection and Measurement of Disease Traits

**FIGURE 1:**
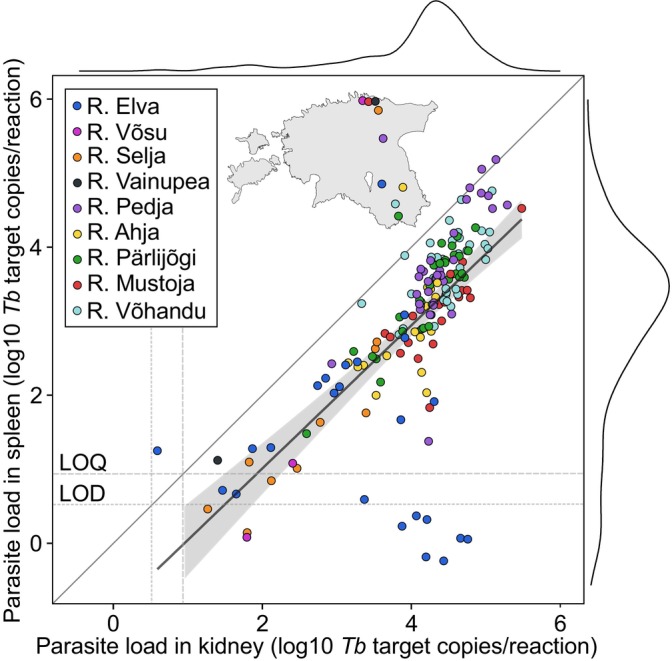
Estimated parasite load (log‐10 *Tetracapsuloides bryosalmonae* (*Tb*) target copies/reaction) from kidney and spleen tissues in 166 young‐of‐the‐year brown trout. The 95% confidence interval is indicated by the grey area beneath the linear regression line. Limit of quantification (LOQ, 9 copies) and limit of detection (LOD, 3.6 copies) are represented by wide and narrow grey dotted lines, respectively. Marginal distributions are depicted with black lines along the top and side figure borders. The insert map indicates the sampling point locations in Estonia.

Young‐of‐the‐year (YOY) wild brown trout (*n* = 238) were collected from August 24 to September 2, 2022 from nine rivers in Estonia (Figure 1, Table 1) using standard electrofishing equipment (experimental fishing permit no. 10–1/22/42–2 issued by Ministry of Regional Affairs and Agriculture of Estonia). YOY were distinguished from older year classes based on size distribution (total length up to ~110 mm, Järvekülg 2003). The fish were euthanised with an overdose of benzocaine, > 250 mg L^−1^ (Caesar & Loretz GmbH, Hilden, Germany) and total length and mass were recorded. Haematocrit level was determined as the red‐blood‐cell‐to‐total volume ratio and sampled from the caudal artery using heparinised micro capillary tubes (diameter 0.5–0.6 mm, Marienfeld, Germany), followed by centrifugation with QBC Capillary Centrifuge (Drucker Diagnostics, USA) for 5 min at 12,250 g. Blood plasma and packed red blood cells were measured to the nearest 0.5 mm using a standard ruler (Debes et al. 2017). The body cavity of the fish was opened with a longitudinal incision from the anal opening to the operculum. The spleen was removed using sterile forceps and placed in 96% ethanol (1.5 mL microcentrifuge tubes) for further DNA extraction and quantification of *Tb*. To investigate the primary PKD symptom, renal hyperplasia, a middle cross‐section of each fish was cut with a sterile scalpel as in Bruneaux et al. (2017). The section was photographed from above on a millimetre paper using a digital camera to calculate the kidney‐to‐body thickness ratio (K/B ratio) as a quantitative measure of renal hyperplasia (Bruneaux et al. 2017; Debes et al. 2017) using open‐source image processing and analysis software ImageJ (Schneider et al. 2012). The cross‐section was stored in 96% ethanol (5 mL screw‐cap centrifuge tubes) for further DNA extraction from the kidney tissue for quantification of *Tb*.

**TABLE 1:** Summary information on sampling sites, *Tetracapsuloides bryosalmonae* prevalence and load.

River	Coordinates (N, E)	No. of YOY* brown trout	*Tb* prevalence (95% CI)		Parasite load *Tb* copies/reaction mean; SD (range)	
			Kidney	Spleen	Kidney	Spleen
Vainupea	59°34′02.4″, 26°15′28.7″	9	0.22 (0.06–0.55)	0.11 (0.02–0.43)	3.5; 8.4 (0–25)	1.5; 4.4 (0–13.2)
Selja	59°23′27.4″, 26°23′30.4″	20	0.75 (0.53–0.89)	0.45 (0.26–0.66)	525; 1120 (0–3462)	54; 146 (0–525)
Võsu	59°33′39.1″, 25°59′13.9″	20	0.15 (0.05–0.36)	0 (0.03–0.30)	17; 58 (0–256)	0.7; 2.7 (0–12)
Ahja	58°08′38.9″, 26°58′28.9″	15	1 (0.80–1)	0.93 (0.70–0.99)	10,938; 7,891 (15–22,367)	886; 1,043 (0–3,298)
Mustoja	59°33′09.9″, 26°10′59.3″	19	1 (0.84–1)	1 (0.84–1)	39,401; 66,636 (4,436‐304,759)	3,261; 7,462 (68.1–33,407)
Mustoja	59°31′48.0″, 26°10′44.4″	20	0.05 (0–0.24)	0.05 (0–0.24)	0.3; 1.2 (0–5.4)	0.1; 0.2 (0–1.1)
Võhandu	57°53′00.1″, 26°44′32.5″	18	1 (0.82–1)	1 (0.82–1)	34,778; 29,842 (2,156‐112,742)	8,178; 10,682 (659–43,858)
Võhandu	57°55′32.7″, 26°45′05.5″	20	1 (0.84–1)	1 (0.84–1)	44,828; 33,177 (7,777‐121,899)	9,943; 12,161 (785–57,500)
Pärlijõgi	57°45′23.6″, 26°45′55.6″	19	1 (0.83–1)	1 (0.83–1)	35,259; 16,239 (3,003‐72,713)	6,433; 4,332 (333–18,353)
Pärlijõgi	59°02′42.1″, 26°14′20.1″	10	1 (0.72–1)	1 (0.72–1)	10,468; 10,458 (393–35,362)	1,665; 3,125 (30–10,434)
Elva	58°08′33.8″, 26°23′51.7″	21	1 (0.84–1)	0.57 (0.36–0.75)	19,150; 15,131 (2,347‐57,310)	127; 431 (0–1,919)
Elva	58°06′54.8″, 26°23′17.7″	17	0.76 (0.53–0.90)	0.71 (0.47–0.87)	898; 1,946 (3.9–8,128)	139; 293 (0–1,216)
Pedja	57°45′23.6″, 26°45′55.6″	11	1 (0.78–1)	1 (0.74–1)	83,739; 55,718 (854–195,132)	51,475; 45,864 (266–152,844)
Pedja	58°56′52.8″, 26°30′25.4″	19	1 (0.83–1)	1 (0.83–1)	19,166; 7,438 (11,852‐37,081)	2981; 1,858 (23–6726)

* Young‐of‐the‐year.

### 2.2 Quantification of Parasite Using qPCR

DNA extractions were conducted in the Fish Genetics Laboratory, Estonian University of Life Sciences. Total genomic DNA from kidney and spleen tissue of each of the 238 brown trout was extracted separately using the QIAamp 96 DNA QIAcube HT kit and the QIAcube HT Instrument for automated nucleic acid purification (QIAGEN, Germany). Whole kidney section and whole spleen or a section of the tissue within highly proliferated organs were used for DNA extractions. Each 96‐well DNA plate included one DNA extraction negative control. DNA concentration was assessed using a NanoDrop 2000 Spectrophotometer (Thermo Scientific, USA), and each extraction was diluted to 20 ng/μl using AE buffer (QIAGEN, Germany). Quantification of *Tb* from extracted kidney and spleen DNA was carried out in a set of three technical replicates using real‐time quantitative PCR (qPCR) on a LightCycler 480 (Roche, Switzerland, 384‐well plates). qPCR analysis was carried out at the Institute of Technology, University of Tartu. The assay utilised a TaqMan probe alongside *Tb*‐specific forward and reverse primers to amplify a 90 bp 18S rDNA sequence of the parasite (PKX18s 1337f: 5′‐CGAACGAGACTTCTTCCTT‐3′, PKX18s 1426r: 5′‐CTTCCTACGCTTTTAAATAGCG‐3′; Hutchins et al. 2018a). The TaqMan/hydrolysis probe PKX18s 1399p (5′‐FAM‐CCCTTCAATTAGTTGATCTAAACCCCAATT‐iQ500‐BHQ‐1‐3′) as described by Hutchins et al. (2018a) was employed.

Each 10 μL amplification reaction contained 4.4 μL of nuclease‐free water, 2 μL of 5x HOT FIREPol Multiplex qPCR Mix (ROX; Solis Biodyne, Estonia), 0.2 μL of 200 nM *Tb* forward and reverse primers, 0.2 μL of 200 nM PKX18s probe and 3 μL of genomic DNA isolated from kidney or spleen tissue (20 ng/μl, total of 60 ng of total tissue DNA per reaction). A tenfold serial dilution of pooled *Tb*‐positive kidney DNA was prepared with concentrations ranging from 40 to 0.004 ng/μl (5 different concentrations) and added to each plate in six technical replicates per concentration, serving as the standard dilution series/positive control. A negative PCR control was included in six replicates on each plate by adding DNase‐RNase‐free water (Solis Biodyne) to the amplification reaction (resulting in 48 PCR negative replicates in total). None of the negative PCR controls showed amplification. In addition, one negative DNA extraction control sample was included on each of the qPCR plates (eight plates in total), with three technical replicates per plate (resulting in 24 replicates in total). Among the eight negative DNA extraction control samples, weak amplification was observed in two samples. In one negative DNA extraction control, one technical replicate out of three showed a Cq (cycle threshold) value of 37.9, while in the other extraction control, two replicates out of three resulted in late amplification (Cq 37.97 and 38.61). Within the positive samples, *Tb* amplified in three replicates out of three in 99.2% of cases (354 out of 357 positive samples).

For estimation of limit of detection (LOD) and limit of quantification (LOQ), a synthetic dilution series was prepared by adding the artificially synthesised *Tb* 18S gene sequence fragment (90 bp; Hutchins et al. 2018a) to one 384‐well plate in a tenfold serial dilution, ranging from 1,806,642.6 to 180.6 copies/reaction (5 different concentrations, with 32 technical replicates per concentration). This was followed by an eight‐fold dilution, reducing the concentration from 180.6 to 22.5 copies/reaction (32 technical replicates). Finally, two 5‐fold dilutions were made, decreasing the concentration from 22.5 to 4.5 to 0.9 copies/reaction (64 technical replicates per concentration). qPCR was performed using the following thermal cycling profile: 95°C for 10 min, followed by 45 cycles of 95°C for 15 s and 60°C for 60 s. All qPCR plates were prepared and run by the same person. Technical replicates were checked for outliers by calculating the standard deviation (SD). Given that parasite load among infected samples can vary dramatically (e.g., Hutchins et al. 2018b), we opted for a standard deviation threshold < 1 when identifying outliers among qPCR technical replicates.

Regression analysis of standard dilution series was performed on each plate to determine qPCR plate efficiencies (Table S1). Additionally, a linear regression analysis of synthetic dilution series was conducted to transform Cq estimates to *Tb* target copies/reaction (*Tb* load). The limit of detection (LOD, 3.6 copies/reaction) and the limit of quantification (LOQ, 9 copies/reaction) were determined based on the synthetic *Tb* 18S rRNA gene dilution series comprising nine dilutions, following the method and R‐script outlined by Merkes et al. (2019) and Klymus et al. (2019). The effective LOD for three technical replicates was 1.1 copies/reaction. Out of the positive samples, 96.6% (345 out of 357 positive samples) amplified above the LOD threshold. For comparative purposes, we also report Cq values in Table S1.

### 2.3 Statistical Analysis

Statistical analyses and figures were created in R version 4.2.2 (R Foundation for Statistical Computing). To evaluate the congruence of parasite load estimates between two tissues, we calculated both Pearson's product moment and rank‐order Spearman's correlation coefficients. We used both raw data (*Tb* target copies/reaction, *n* = 191) and log10 transformed parasite load data (log10 *Tb* target copies/reaction, *n* = 166). The log10 transformation was applied to address the significant right skewness in the data, aiming to make its distribution more closely approximate normality (data not shown). Since it is not possible to log10 transform zero values, the transformed data consisted of only individuals in which parasite was detected in both tissues (*n* = 166). The non‐parametric Wilcoxon signed‐rank test was used to compare parasite prevalence between different tissues in the nine brown trout populations. Earlier studies have demonstrated a non‐linear relationship between *Tb* load and disease traits (e.g., Debes et al. 2017; Lauringson et al. 2021). Therefore, we aimed to further explore the dynamics between parasite load and disease symptoms by carrying out segmented regression analysis (also known as broken‐line models) using the R package ‘Segmented’ (Muggeo 2008). More specifically, we used segmented regression line analysis to determine at what parasite load level (log10 transformed) for each tissue measured health parameters (renal hyperplasia and haematocrit) started to deteriorate faster. This is known as a breakpoint in segmented regression models and refers to the value of the predictor (parasite load in this case) at which the relationship between the independent and dependent variables (renal hyperplasia and haematocrit) changes in a piecewise linear manner. Davies' test was employed to assess the significance of slope changes.

## 3 Results

### 3.1 Parasite Load and Prevalence Across Tissues

Out of the 238 specimens sampled from nine rivers, *Tb* was detected in 191 individuals (Table 1). Detection rates were higher in kidney samples (190/191; 99.5%) than in spleen samples (167/192; 87.5%, Table S1).

In most cases, amplification was observed in both tissues. However, in a single specimen, low parasite concentration was detected exclusively in the spleen (1.1 *Tb* target copies/reaction), while 23 individuals showed amplification only in the kidney (range: 4.5–50,370 *Tb* target copies/reaction). All positive kidney amplifications were above LOD (3.6 *Tb* target copies/reaction), while 15 spleen samples fell below LOD. Additionally, nine kidney and 38 spleen *Tb* amplifications fell below LOQ (9 *Tb* target copies/reaction). On average, the kidney showed 4.1‐fold higher parasite abundance than the spleen (25,777 vs. 6259 *Tb* target copies/reaction).

Average parasite prevalence in the studied rivers was 73.7% (kidney) and 63.7% (spleen), though the difference in parasite prevalence between tissues was not significant (*p* = 0.062, Wilcoxon signed‐rank test).

Parasite load between tissues showed significant positive correlation. For untransformed data, Pearson's *r* = 0.60 (*p* = 1.15 × 10^−19^) and Spearman's *ρ* = 0.78 (*p* = 3.01 × 10^−40^). For log10 transformed data, Pearson's *r* = 0.71 (*p* = 2.49 × 10^−26^) (Figure 1).

### 3.2 Relationships Between Parasite Load and Disease Symptoms

**FIGURE 2:**
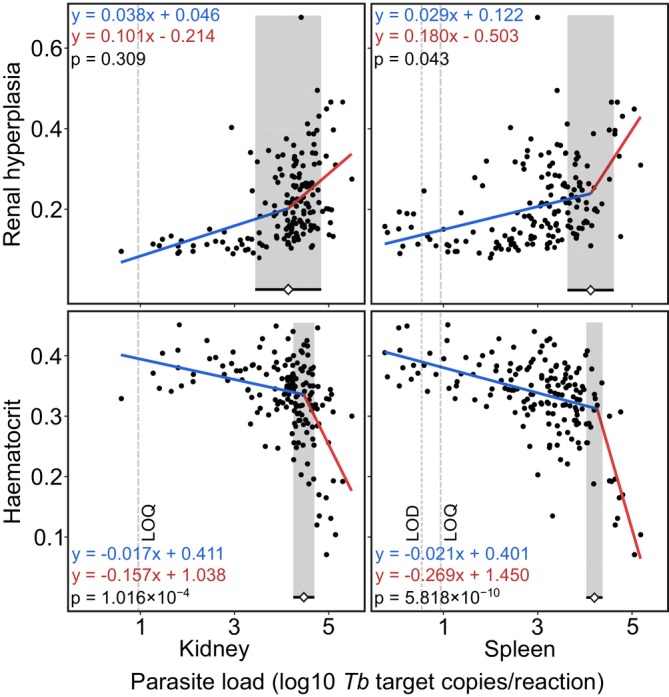
Segmented linear regression relationships between parasite load (log10 *Tetracapsuloides bryosalmonae (Tb)* target copies/reaction) and disease traits (renal hyperplasia and haematocrit). 95% confidence intervals of breakpoints are marked with the grey area, with the white diamond marking the breakpoint estimate. LOQ (9 copies) and LOD (3.6 copies) are shown with wide and narrow grey dotted lines, respectively. The first slope at lower parasite load levels is marked in blue, and the second slope for higher parasite load levels is marked in red, with each subfigure displaying the respective regression formulas and Davies' test.

Segmented regression analysis indicated a significant breakpoint in relationships between parasite load and both, renal hyperplasia and haematocrit in three out of the four tissue‐specific analyses, reflecting accelerated health deterioration beyond specific parasite load thresholds. The estimated breakpoint confidence intervals were wider for renal hyperplasia than for haematocrit for both tissues (Figure 2).

For haematocrit, breakpoints were estimated at 4.263 log10 *Tb* target copies/reaction (95% CI: 4.037–4.374) for spleen and 4.471 log10 *Tb* target copies/reaction (95% CI: 4.255–4.687) for kidney. Slopes differed significantly above and below these thresholds for both tissues (*p* = 5.818 × 10^−10^ and 1.016 × 10^−4^, respectively; Davies' test). For renal hyperplasia, breakpoints were estimated at 4.126 log10 *Tb* target copies/reaction (95% CI: 4.037–4.374) for spleen and 4.14 log10 *Tb* target copies/reaction (95% CI: 3.456–4.824) for kidney. However, slopes differed significantly above and below estimated threshold only for spleen (*p* = 0.043, Davies' test).

## 4 Discussion

This study evaluates the suitability of spleen tissue as an alternative to kidney tissue for detecting and quantifying *Tetracapsuloides bryosalmonae* (*Tb*) in juvenile brown trout. By assessing parasite prevalence in both tissues across a large number of individuals, we aimed to clarify the relationship between parasite load and key clinical indicators of proliferative kidney disease (PKD) ‐ renal hyperplasia and anaemia. Our findings revealed a strong positive correlation between the two tissues, despite *Tb* being more than four times less abundant in the spleen. Below, we discuss the usefulness and limitations of the spleen as an alternative to the kidney tissue for estimating *Tb* load, prevalence, and its relationships with PKD symptoms in juvenile brown trout.

Histological studies of PKD have shown that well‐vascularised extra‐renal organs, such as the spleen, often exhibit pathological changes similar to those observed in the kidney (Kent 2002). Consistent with this, our analyses demonstrated a moderate‐to‐strong correlation in parasite load between the spleen and kidney. Nevertheless, reflecting the kidney's role as the primary site of parasite development (Okamura et al. 2011), the parasite load in the kidney was, on average, 4.1 times higher than in the spleen. Despite this disparity, the estimated parasite prevalence did not differ between the tissues across nine river populations, as determined by paired Wilcoxon signed‐rank test. This is likely caused by the lack of statistical power associated with the non‐parametric test. Nevertheless, parasite prevalence estimates in the spleen align well with our previous studies in the same rivers, with some populations showing 100% prevalence in both tissues (e.g., Dash and Vasemägi 2014; Debes et al. 2017).

Standard molecular assessment of the PKD agent typically involves sampling posterior, anterior or whole kidney sections (e.g., Bailey et al. 2017; Vasemägi et al. 2017; Strepparava et al. 2018; Gorgoglione et al. 2019; Philpott et al. 2025). This process usually includes in situ dissection of fresh kidney tissue or fixing the entire or a part of a fish in a fixative, followed by mechanical segregation and lysing the kidney tissue for DNA extraction. However, due to the kidney's deep position within the body cavity and high vascularisation, kidney dissection can be time‐consuming, particularly when processing large sample sizes. In contrast, the spleen is more accessible and offers dense lymphoid and red pulp tissues, facilitating easier and faster sample collection.

Our findings suggest that, while parasite load in the spleen is typically lower than in the kidney, the spleen is still a viable tissue for *Tb* detection and quantification. Notably, we detected the parasite in both tissues of YOY fish that did not exhibit clinical PKD signs, suggesting early parasite presence in both the kidney and spleen. This aligns with earlier work by Longshaw et al. (2002), who reported the presence of *Tb* in the kidney and spleen of rainbow trout at 4 weeks post exposure, notably when the clinical PKD symptoms were absent. Therefore, in cases of low parasite load or asymptomatic PKD, the spleen may serve as a useful additional diagnostic tissue. Furthermore, Soliman et al. (2017) detected *Tb* in the spleen and other lymphoid organs of brown trout with subclinical PKD 5 years post‐infection. Thus, analyses of spleen parasite load are expected to provide valuable insights into *Tb* infections during the subclinical phase and potential (re)infections in older fish. Unlike juveniles, which typically exhibit consistent *Tb* infection across the kidney, parasite DNA is predominantly detected in the middle and posterior kidney sections within adult trout (Dash and Vasemägi 2014). This suggests that only sporogonic stages in the excretory section persist long‐term, while extrasporogonic stages in the anterior kidney are likely eliminated (Dash and Vasemägi 2014). Notably, parasite detection in the anterior kidney of adult sea trout indicates the possibility of infections or re‐infections in later life stages (Lauringson et al. 2023). Future research could investigate whether anadromous fish, which are not exposed to the parasite during their marine phase, can eliminate *Tb* from the spleen after initial exposure.

Despite its accessibility and ease of sampling, spleen tissue presents some limitations. The parasite load in the spleen is consistently lower than in the kidney, which may reduce sensitivity in cases of early or low‐intensity infections. Additionally, spleen‐based detection may not fully capture the parasite's developmental stages or infection status, as the spleen is not the primary site of parasite proliferation. Furthermore, we identified a group of samples from the river Elva, where spleen parasite load was considerably lower in relation to kidney. This may reflect local differences in infection dynamics, including the possibility that extrasporogonic parasite stages have been cleared from the spleen, similar to the kidney (Feist and Longshaw 2006). However, without longitudinal parasite load data and histopathological analyses, the underlying cause why these specimens deviate from the typical parasite load relationship between the two tissues remains unclear. Furthermore, since this study focused exclusively on YOY fish, it is unknown whether the strong positive correlation in parasite load between spleen and kidney persists in older year‐classes.

To further investigate the relationship between parasite burden and disease progression, we used segmented regression analysis to identify parasite load thresholds beyond which health parameters (renal hyperplasia and haematocrit) deteriorate more rapidly. Segmented regression is a robust tool for detecting shifts in variable relationships (Muggeo 2008) and has been widely applied in studies of various infectious diseases (e.g., Gebski et al. 2012; Funk et al. 2013). In our study, breakpoint analysis revealed distinct transitions in the relationship between parasite load and haematocrit for both spleen and kidney tissues. Steeper post‐breakpoint slopes indicated an accelerated impact of parasite proliferation on anaemia at high parasite loads (4.263 and 4.471 log10 *Tb* target copies/reaction for spleen and kidney, respectively). However, the lower breakpoint estimates and steeper post‐break slope for spleen tissue suggest tissue‐specific differences in how parasite burden correlates with haematocrit decline.

For renal hyperplasia, the breakpoint for spleen tissue was marginally significant (*p* = 0.043) and non‐significant (*p* = 0.308) for kidney tissue. The lower breakpoint estimates suggest that renal hyperplasia manifests earlier in PKD progression than anaemia, corroborating earlier research (Bruneaux et al. 2017; Debes et al. 2017; Lauringson et al. 2021). Together, these results highlight the value of segmented regression in identifying threshold‐dependent deterioration in PKD‐related health metrics with increasing parasite burden, offering useful insights into disease progression and host–pathogen dynamics. Additionally, breakpoint analysis could help identify inter‐population differences in host tolerance to *Tb*. However, to apply this method effectively, future studies should standardise and characterise temporal disease progression across systems, as PKD dynamics can vary significantly due to environmental and genetic factors. It is also important to acknowledge that breakpoint estimates can be influenced by data transformation methods; therefore, interpretations should be approached with caution.

In summary, this study demonstrates that spleen tissue provides comparable diagnostic value to kidney tissue for detecting and quantifying *Tetracapsuloides bryosalmonae* in juvenile salmonids. Our findings also highlight the potential added value of tracking parasite load across multiple tissues, which can yield valuable insights into disease progression, immune responses and pathogen behaviour. Ultimately, this study underscores the importance of optimised multi‐tissue diagnostic strategies to enhance understanding and management of parasitic infections across diverse host species and ecological settings.

## Author Contributions

**Magnus Lauringson:** methodology, investigation, analysis, data curation, writing – original draft. **Lilian Pukk:** investigation, analysis, datacuration, writing – review and editing. **Siim Kahar:** investigation, writing – review and editing. **Oksana Burimski:** analysis, writing – review and editing. **Riho Gross:** investigation, writing – review and editing. **Veljo Kisand:** writing – review and editing. **Anti Vasemägi:** methodology, investigation, analysis, data curation, writing – original draft.

## Ethics Statement

Fish sampled in the study were euthanised in accordance with the principles described in Directive 2010/63/EU of the European Parliament and of the Council of 22 September 2010 on the protection of animals used for scientific purposes.

## Conflicts of Interest

The authors declare no conflicts of interest.

## Supporting information


**Table S1** Breakpoint estimates based on segmented line analysis, detection rates and detailed qPCR parameters of kidney and spleen tissues. Kidney qPCR plates consisted of more plates and additional samples (excluded from calculations), which were used for another study; therefore, plates 4 and 5 are marked as N/A for spleen samples. *Calculations were performed with the first four 10‐fold serial dilution concentrations.

## Data Availability

All datasets generated and analysed during the current study are available from the corresponding author upon request.
