# Supplementary material for: Spleen as an Alternative Tissue for Estimating Tetracapsuloides bryosalmonae Load, Prevalence and Its Relationship With Proliferative Kidney Disease in Brown Trout
Source: J Fish Dis. 2025 Jun 5;49(1):e14148. doi: 10.1111/jfd.14148 (PMC12667013; doi:10.1111/jfd.14148)
Supplement: Supplementary file 1 — Table S1 Breakpoint estimates based on segmented line analysis, detection rates and detailed qPCR parameters of kidney and spleen tissues. Kidney qPCR plates consisted of more plates and additional samples (excluded from calculations), which were used for another study; therefore, plates 4 and 5 are marked as N/A for spleen samples. *Calculations were performed with the first four 10‐fold serial dilution concentrations. [file JFD-49-e14148-s001.docx]

**Spleen as an alternative tissue for estimating *Tetracapsuloides bryosalmonae* load, prevalence and its relationship with proliferative kidney disease in brown trout**

**Authors:** Magnus Lauringson^1^, Lilian Pukk^1^, Siim Kahar^1^, Oksana Burimski^1^, Riho Gross^1^, Veljo Kisand^2,3^, Anti Vasemägi^1,4^

**Appendices**

**Table S1.** Break-point estimates based on segmented line analysis, detection rates and detailed qPCR parameters of kidney and spleen tissues. Kidney qPCR plates consisted of more plates and additional samples (excluded from calculations), which were used for another study, therefore plate 4 and 5 are marked as N/A for spleen samples. *Calculations were performed with first four ten-fold serial dilution concentrations.

| **Parameter** | **Kidney** | **Spleen** |
| --- | --- | --- |
| Renal hyperplasia break-point (log10 *T.b* target copies/reaction) | 4.14 | 4.126 |
| Haematocrit break-point (log10 *T.b* target copies/reaction) | 4.471 | 4.263 |
| Positive *T.b* amplifications n, total n | 190, 238 | 167, 238 |
| *T.b* detection rate %* | 99.5 | 87.5 |
| No. of *T.b* amplifications above LOQ, above LOD | 182, 190 | 152, 156 |
| Average and median log10 *T.b* target copies/reaction | 4.41, 4.23 | 3,85, 1.43 |
| 25 % and 75 % percentile log10 *T.b* target copies/reaction | 3.53, 4.52 | 1.39, 1.47 |
| Average and median Cq value | 25.2, 23.7 | 28.1, 27.1 |
| 25 % and 75 % percentile Cq value | 22.7, 26.1 | 25.2, 29.9 |
| Mean SD for technical replicate Cq values | 0.098 | 0.136 |
| Median SD for technical replicate Cq values | 0.051 | 0.087 |
| 25 percentile for technical replicate Cq values | 0.030 | 0.055 |
| 75 percentile for technical replicate Cq values | 0.095 | 0.157 |
| Plate 1 amplification efficiency % | 91.45* | 95.06 |
| Plate 2 amplification efficiency % | 92.16 | 91.88 |
| Plate 3 amplification efficiency % | 92.25* | 90.71 |
| Plate 4 amplification efficiency % | 93.20 | N/A |
| Plate 5 amplification efficiency % | 91.68* | N/A |
| Plate 1 *r*^2^of calibration curve | 0.999 | 0.998 |
| Plate 2 *r*^2^ of calibration curve | 0.993 | 0.998 |
| Plate 3 *r*^2^ of calibration curve | 0.999 | 0.998 |
| Plate 4 *r*^2^ of calibration curve | 0.997 | N/A |
| Plate 5 *r*^2^ of calibration curve | 0.999 | N/A |
